# Analysis of misoprostol and chlorhexidine policy gains in Pakistan: the advocacy experience of Mercy Corps Pakistan

**DOI:** 10.1186/s12961-015-0037-4

**Published:** 2015-11-25

**Authors:** Zahida Sarwar, Andrea Cutherell, Arif Noor, Farah Naureen, Jennifer Norman

**Affiliations:** Policy Strategy & Planning Unit, Department of Health, Government of Punjab, Lahore, Pakistan; Mercy Corps Pakistan, Islamabad, Pakistan; Mercy Corps, Washington, DC USA

**Keywords:** Advocacy, Chlorhexidine, Maternal, Misoprostol, Policy

## Abstract

While Pakistan has made progress toward achieving Millennium Development Goal 5 for maternal health, it is unlikely to achieve the target; further, it is also not on track for Millennium Development Goal 4 regarding child health. Two low-cost, temperature stable and life-saving drugs, misoprostol and chlorhexidine, can respectively avert maternal and newborn deaths, and are particularly pertinent for poor and marginalized areas which bear the brunt of maternal and newborn deaths in Pakistan. In response, Mercy Corps led focused advocacy efforts to promote changes in policies, protocols, and regulatory environments for misoprostol (2012–2014) and for chlorhexidine (2014). These short-duration advocacy projects facilitated significant policy gains, such as inclusion of misoprostol and chlorhexidine into province-specific essential drug lists, development and endorsement of clinical protocols for the two drugs by provincial health departments, inclusion of misoprostol into pre-service training curriculum for several health cadres, and application for registration of chlorhexidine (at the concentration required for newborn care) by two pharmaceutical companies. These results were achieved by a consultative and evidence-based process which generated feedback from community members, program implementers, and policymakers, and ultimately put the government in the driver’s seat to facilitate change. Community Action Dialogue forums were linked with provincial-level Technical Working Groups and Provincial Steering Committees, who passed on endorsed recommendations to the Health Secretary. The key factors which facilitated change were the identification of champions within the provincial health departments, prioritization of relationship building and follow-up, focus on concrete advocacy aims rather than broad objectives, and the use of multi-stakeholder forums to secure an enabling environment for the policy changes to take root. While these advocacy initiatives resulted in significant policy changes in Pakistan’s devolved health system, to ensure these policy changes have an impact on health outcomes, Pakistan should focus on the scale-up of appropriate use of chlorhexidine and misoprostol. Further, future policy initiatives in Pakistan should make use of similar multi-stakeholder policy forums, while ensuring a third party to facilitate the process so that civil society and community voices are not lost in the policy development discussion.

## Background

While Pakistan has made progress toward achieving Millennium Development Goal 5 for maternal health, it is unlikely to achieve the stipulated target; further, it is also not on track to achieve Millennium Development Goal 4 regarding child health. With a maternal mortality of 170 per 100,000 live births, Pakistan is among the 10 countries contributing 58% of all maternal deaths reported in 2013 [1]. Poor maternal health outcomes are directly linked to poor child health outcomes, particularly for infants (i.e. under age 1) and newborns (i.e. 28 days or younger). Pakistan has the third highest number of newborn deaths in the world (194,000 in 2010) [2].

The leading cause of maternal deaths (27%) in Pakistan is postpartum haemorrhage (PPH) [3], a condition which is easily preventable or treatable through proper use of uterotonic drugs (drugs which contract the uterus to reduce bleeding). Ideally, PPH should be prevented/treated through the participation of skilled birth attendants (SBA) who can administer oxytocin through a reliable cold chain. However, only 52% of births in Pakistan are attended by a SBA, only a portion of which have access to a reliable cold chain [4]. In the absence of a reliable cold chain and until SBAs are widely accessible, misoprostol (a temperature-tolerant drug in tablet form) can make significant contributions toward reducing maternal mortality by preventing and treating PPH [5–9].

Nearly two-thirds of all child deaths in Pakistan occur during the newborn period, with 74% of newborn deaths taking place during the first week of life [4]. Thus, improving access to lifesaving drugs and skills immediately following birth and the days after is critical to averting newborn deaths. Worldwide, up to 30% of newborn deaths are attributable to sepsis, a condition which could be prevented through proper umbilical cord care [10]. Chlorhexidine (specifically 7.1% chlorhexidine digluconate delivering 4% active chlorhexidine) is a temperature-tolerant low-cost drug which can reduce newborn mortality rates by up to 23% [11]. It can be administered to the umbilical stump to prevent sepsis by any health care provider or even family members.

These two low-cost, temperature-stable, and life-saving drugs, misoprostol and chlorhexidine, are particularly pertinent for poor and marginalized areas, which bear the brunt of maternal and newborn deaths in Pakistan.

## Mercy Corps Pakistan response

In response to the challenges facing maternal and newborn survival, Mercy Corps led focused advocacy efforts with the goal of promoting changes in policies, protocols, and regulatory environments for misoprostol between 2012 and 2014 and for chlorhexidine in 2014. At the time of the initiatives, misoprostol was widely available but underutilized for the purpose of PPH prevention and treatment. Chlorhexidine, at the concentration required for newborn cord care, was not locally produced nor available in the market. Both advocacy initiatives targeted the provincial departments of health. After the devolution of the federal health system in 2011, the departments have been responsible for the development and implementation of health policies within their province/region. Misoprostol advocacy work took place in two provinces (Balochistan and Khyber Pakhtunkhwa) and two regions (Azad Jammu Kashmir and the Federally Administered Tribal Areas). Chlorhexidine advocacy work targeted three provinces (Balochistan, Punjab, and Sindh) and two regions (Azad Jammu Kashmir and Gilget-Baltistan). It should be noted that Balochistan has some of the poorest health outcomes in Pakistan, for example, with an estimated maternal mortality of 785 per 100,000 live births [3].

### Policy gains

The advocacy projects were of short duration, lasting 1–2 years, and facilitated significant policy gains. In summary, misoprostol was included in province-specific essential drug lists; clinical protocols for appropriate use of misoprostol in community and facility settings were developed and endorsed by provincial health departments; and the pre-service training curriculum was revised to include misoprostol for key health cadres including nurses and Lady Health Visitors (facility based), Lady Health Workers (LHWs; community based), and Community Midwives (community based). With regards to chlorhexidine, clinical guidelines for multiple application of chlorhexidine until 7 days immediately after birth or until the cord stump separates were approved by provincial health departments; it was included in provincial essential drug lists and purchase lists of the Executive District Officer Health (government’s lead representative for the health system at the district level); it was included in Community Midwives birthing kits and LHW service lists; and two pharmaceutical companies (MediSearch and ZAFA) applied for the registration of 7.1% chlorhexidine digluconate delivering 4% active chlorhexidine to the Drug Regulatory Authority.

### Advocacy pathways

To achieve these results, Mercy Corps worked in partnership with the provincial departments of health and other key stakeholders (for example, the misoprostol advocacy initiative was implemented in partnership with Khwendo Kor, a local NGO). A consultative process which put the government in the driver’s seat was facilitated to determine which policy changes to make. The consultative strategy aimed to contextualize global best practices for misoprostol and chlorhexidine for Pakistan, on the basis of feedback and inputs from community members, program implementers, and policymakers. Mercy Corps followed a three-step process to achieve its policy aims.

#### Consolidation of evidence

In both initiatives, Mercy Corps first commissioned a desk review to look at the global, regional, and local evidence available for the particular drug. This was followed by field consultations in the form of key informant interviews and focus group discussions to understand perceptions, availability, and use of the drugs. The desk review and field consultations were combined into a situational analysis which was widely disseminated to all stakeholders.

#### Presentation of evidence

To facilitate a multi-stakeholder process which ensured that community voices were reflected in policy discussion, Mercy Corps supported three tiers of forums. First, Community Advocacy Dialogue Forums engaged local NGOs, key influencers, community and district representatives, and stakeholders. Feedback was gained on strategies to influence provincial level policy. The participant’s commitment to implementing policies was also endorsed. The feedback from the Community Advocacy Dialogue Forums was shared with provincial level Technical Working Groups (TWG), which reviewed the evidence (e.g. from the situational analysis) and the feedback. Recommendations were provided by the TWGs to the Provincial Steering Committees (PSCs) on the specific policies necessary to endorse. The PSCs were chaired by the Deputy General Health Services who passed on endorsed recommendations to the Health Secretary. The target audiences for the advocacy efforts therefore included (1) policymakers at the provincial level, including but not limited to women parliamentarians, the Minister for Health, the Health Secretary, the Director General for Health Services, and provincial Programme Managers for the Provincial Programme for Family Planning and Primary Health Care and the Provincial Programme for Maternal, Newborn and Child Health (MNCH), which included representatives of the Society of Obstetricians and Gynaecologists of Pakistan, the Pakistan Nursing Council, the Pakistan Medical Association, and the Pakistan Paediatric Association; (2) policy implementers at the district level, including but not limited to the Executive District Officer (Health), District Coordinators for LHWs and MNCH programmes, public and private healthcare providers, owners of private pharmacies/drug stores, and obstetricians and gynaecologists; and (3) opinion leaders/champions, including but not limited to locally elected parliamentarians/politicians, civil society and community-based organizations, journalists, religious leaders, tribal chiefs, LHWs and other community health workers such as traditional birth attendants, and women of reproductive age.

#### Consensus building

Through these tiers of multi-stakeholder forums combined with one-on-one advocacy meetings, Mercy Corps facilitated a process whereby the government, in consultation with the leading organizations and individuals in MNCH at the provincial level, came to their own conclusions about which policies should be endorsed. Mercy Corps positioned itself as a facilitator, not the leader, to ensure the conclusions were truly driven by the government and thus sustainable after the advocacy initiatives came to a close.

## What facilitated change?

There were several key factors which facilitated change. First, Mercy Corps identified champions within the provincial departments of health to spearhead the advocacy efforts from within the government. Second, Mercy Corps emphasized relationship building and follow-up which consumed the majority of the time that staff dedicated to this project. Third, the initiatives’ focus on specific, concrete advocacy aims, rather than broad objectives, helped the committees to target their efforts and create change in a particular area. Finally, and perhaps most importantly, multi-stakeholder forums, which brought together influential players from all backgrounds, was paramount to securing an enabling environment for the policy changes to be proposed and eventually take root.

## Conclusion

While these advocacy initiatives resulted in significant policy changes in Pakistan’s devolved health system, the changes will only have an impact on MNCH outcomes if they are implemented in a timely way with sufficient support and oversight to ensure quality of service delivery. Thus, the battle is only half-won and the attention of the MNCH community in Pakistan should focus on the scale-up of appropriate use of chlorhexidine and misoprostol, in addition to the multiple other priorities in the sector. Further, several of the provincial departments of health have constituted TWGs and PSCs within the government; future policy initiatives in Pakistan should make use of these forums. It remains critical for a third party to facilitate the process to ensure civil society and community voices are not lost in the policy development discussion.

## Abbreviations

LHWs: Lady health workers
MNCH: Maternal, newborn and child health
PPH: Postpartum haemorrhage
PSCs: Provincial Steering Committees
SBA: Skilled birth attendants
TWG: Technical working groups
